# 

**Published:** 1883-02

**Authors:** 


ORIGINAL COMMUNICATIONS:
Medical Clinic. Prof. Jas. T. Whittaker, M.D., Cincinnati .... 65
Trephining for Depressed Fracture in Vertex of Skull. Parker Syms, M.D. .. 72
The Germ Theory of Dental Caries. Dr. C. T. Stockwell, Conn .... 73
Wounds of Eye-Ball. Peter A. Callan, M. D .... 79

ORIGINAL TRANSLATIONS AND ABSTRACTS:
Chronic Diphtheria of the Pharynx .... 81
Electrical Phenomena in the Mouth. Dr. Willoughby D. Miller, of Berlin .. 86
Cerebral Tumor .... 90
On the Cure of Epilepsy by Ligature of the Vertebral Arteries .... 91
The Muse in Medicine .... 91

SELECTIONS:
The Influence of Constitutional Diathesis on the Development of Disease Germs .... 92
An Experience with the New Anæsthetic—Chloroform, Ether and Absolute Alcohol .... 97
The Influence of the Trigeminus Nerve on the Organs of Hearing .... 100

EDITORIAL:
Medical Literature and Medical Journals .... 101
Microscopic Organisms and Antisepsis .... 103
Relaxation for Dentists .... 104
Gold Alloy .... 109
Sponges for Dentists' Use .... 110

CURRENT NEWS AND OPINION:

New York Pathological Society .... 111

New York Academy of Medicine .... 111

Cockroach Tea .... 112

BIBLIOGRAPHICAL .... 114

OBITUARY:
George M. Beard, A.M., M.D .... 118
Marshall H. Webb, D.D.S .... 119

EXTRACTS:
Radical Cure of Popliteal Aneurism .... 121
Lesions of the Teeth in Locomotor Ataxy .... 122
A Convenient and Delicate Test for Albumen .... 122
The Mechanical Treatment of Neuralgia .... 123
Placenta Prævia .... 123

POPULAR SCIENCE DEPARTMENT:
Parasites in the Pork Supply of Montreal .... 124
A Medical Opinion of the Electric Light .... 127
The First Telephone .... 127

